# Mechanical Milling-Assisted Spark Plasma Sintering of Fine-Grained W-Ni-Mn Alloy

**DOI:** 10.3390/ma11081323

**Published:** 2018-07-31

**Authors:** Yanlin Pan, Daoping Xiang, Ning Wang, Hui Li, Zhishuai Fan

**Affiliations:** 1 State Key Laboratory of Marine Resource Utilization in South China Sea, Hainan University, Haikou 570228, China; yanlinpan2014@163.com (Y.P.); wangn02@foxmail.com (N.W.); lihui1285@126.com (H.L.); zsfan2018@163.com (Z.F.); 2 State Key Laboratory for Advanced Metals and Materials, University of Science and Technology Beijing, Beijing 100083, China

**Keywords:** W-6Ni-4Mn alloys, mechanical milling, spark plasma sintering, transmission electron microscopy, mechanical property

## Abstract

Fine-grained W-6Ni-4Mn alloys were fabricated by spark plasma sintering (SPS) using mechanical milling W, Ni and Mn composite powders. The relative density of W-6Ni-4Mn alloy increases from 71.56% to 99.60% when it is sintered at a low temperature range of 1000–1200 °C for 3 min. The spark plasma sintering process of the alloy can be divided into three stages, which clarify the densification process of powder compacts. As the sintering temperature increases, the average W grain size increases but remains at less than 7 µm and the distribution of the binding phase is uniform. Transmission electron microscopy (TEM) observation reveals that the W-6Ni-4Mn alloy consists of the tungsten phase and the γ-(Ni, Mn, W) binding phase. As the sintering temperature increases, the Rockwell hardness and bending strength of alloys initially increases and then decreases. The optimum comprehensive hardness and bending strength of the alloy are obtained at 1150 °C. The main fracture mode of the alloys is W/W interface fracture.

## 1. Introduction

The kinetic energy penetrators mainly include depleted uranium alloys (DU) and tungsten heavy alloys (WHAs) [1]. DU warheads are prone to adiabatic shear failure in armory, therefore, warheads exhibit high penetration depth. However, radioactive uranium causes serious environmental pollution; as such, WHAs have been extensively investigated. Under high rate loading, the materials that have been incorporated into the bands can momentarily reach very high temperatures [2], which is called “adiabatic shear bands” (ASBs), and adiabatic shear resistance can hinder the formation of ASBs. For conventional W-Ni-Fe alloys, a warhead easily forms a mushroom-like head during armor piercing because these alloys are insensitive to adiabatic shear resistance [3,4]. Thus, the penetration capability decreases by 8–10%. Thermal conductivity plays a major role in the formation of adiabatic shear band. In the 1990s, Bose et al. [5] found that the adiabatic shear performance of WHAs greatly increases when Mn, not Fe, is used because of the low thermal conductivity of Mn. Thus, the penetration depth is not reduced. In recent years, Young and co-workers [6] have discussed the effects of manganese on WHAs. Therefore, W-Ni-Mn alloy can be used as a potential alternative of depleted uranium alloy materials.

The W-Ni-Mn alloys are usually prepared via traditional liquid-phase sintering methods. The effects of various factors such as atmosphere, temperature and content on the densification and properties of W-Ni-Mn alloy have been studied [5,6,7,8,9,10,11,12,13]. However, the residual air in the formed green body and the long time of traditional liquid phase sintering will easily lead to the oxidation of Mn and the growth of W grain size which is not favorable to the improvement of sintered density and mechanical properties [8].

Recently, in order to overcome the defects of traditional liquid sintering, the SPS technique has been successfully used to prepare WHAs [14,15]. SPS is a newly arisen sintering technique, during the sintering process, a high densification rate is favored whereas coarsening induced by surface diffusion is minimized, and then grain growth can be suppressed [14]. Moreover, it is stated that SPS can offer other benefits, such as partial oxide film elimination, adsorbed gas release and surface activation of powder particles [14]. In previous study [13], we found that the densification temperature of spark plasma sintered W-Ni-Mn alloy by simple mixing starting powders was as high as 1250 °C. For fabricating high-density W-Ni-Mn alloy with finer W grain at low temperature, mechanical milling (MM) was applied to the pretreatment of starting powders in this study, and the densification behavior of the alloys has been discussed especially by the sintering curve. Certainly, microstructure and mechanical properties of the alloys were also investigated.

## 2. Experimental

The starting powders were W powder (>99.5 wt.%) with average particle sizes of 4 μm (Zhuzhou Jingzuan Cemented Carbide Co., Zhuzhou, China), Ni powder (>99.5 wt.%) with average particle size of 1 μm (Shanghai Aladdin Bio-Chem Technology Co., Shanghai, China), and Mn powder (>99.5 wt.%) with average particle size of 2.8 μm (Ara Elsa Chemical Co., Shanghai, China). The mass ratio of W-Ni-Mn was 90:6:4. The starting powders were mechanically milled firstly by high-energy ball milling in Ar atmosphere. The ball-to-powder weight ratio was set at 5:1, and cemented carbide balls were used as grinding media. The milling speed was 340 rpm and the milling time was 20 h. Next the milled powders of 72.10 g in each experiment were placed into a cylindrical graphite die in an Ar-filled glove box, and then sintered on a LABOX^TM^−3010K spark plasma sintering system (SINTER LAND INC., Nagaoka, Japan) in a vacuum (≤6 Pa) at a temperature range of 1000 to 1200 °C for 3 min with a heating rate of 100 °C/min. In the process of sintering, the axial pressure of 30 MPa remains constant, which is a commonly used pressure value during SPS [14,16]. And the infrared radiation thermometer (CHINO Co., Tokyo, Japan) is used for measuring sintering temperature of the samples thorough a small hole with several millimeter depths on the die wall. The samples were cooled to room temperature and then taken out from the SPS system. Finally, the sintered W-Ni-Mn alloys with a height of 6 mm and a diameter of 30 mm were fabricated.

The phases and particle size distribution of the composite powders are determined by an X-ray diffraction (XRD) instrument (D8 Advance, Bruker Co., Karlsruhe, Germany) and a laser particle size analyzer (MS2000, Malvern Panalytical, Malvern, UK) respectively. The density of the sintered alloys was measured by Archimedes’ principle. In view of the fact that open porosity may coexist with closed porosity in the sintered alloys, the samples are sealed in advance by paraffin wax. Meanwhile, in order to more accurately measure the density of bulk materials, the sintered alloys are soaked in the water for several minutes until the measured weight value reaches steady state. The hardness was measured by a Rockwell hardness tester (HRS–150, Laizhou Huayin Instrument Co., Laizhou, China). The bending strength was tested at room temperature using a three-point bending test method by a universal testing machine (AGS–X/10KN, SHIMADZU Co., Kyoto, Japan), and the test samples were 6.5 mm wide and 5 mm thick, with a fixture span of 15 mm and a load rate of 0.05 mm/min. The microstructure and fracture morphology of the sintered alloys were observed under a field emission scanning electron microscope (S–4800, Hitachi, Tokyo, Japan). The fine structure of the sintered alloys was investigated using a field emission transmission electron microscope (Tecnai G2 F20, FEI Co., Hillsboro, OR, USA).

## 3. Results and Discussion

### 3.1. Characterization of W-6Ni-4Mn Composite Powders

**Figure 1:**
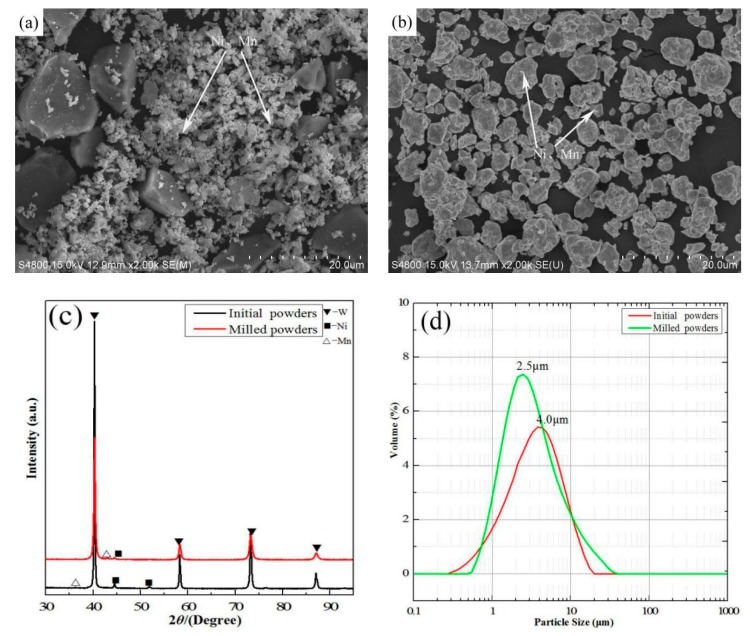
The characterization of W-6Ni-4Mn composite powders: (**a**) starting powders, (**b**) after mechanical alloying, (**c**) XRD patterns, and (**d**) particle size distribution curves.

The characterizations of the W-6Ni-4Mn composite powders are illustrated in Figure 1. The SEM micrographs of the W-Ni-Mn composite powders are presented in Figure 1a,b. Without mechanical alloying, the starting powders exhibit large irregular tungsten particles with a small amount of nickel and manganese micro-particles (Figure 1a). Then the composite powders were dramatically changed in particle sizes after 20 h of milling, as shown in Figure 1b. The XRD pattern of the initial powders and the mechanically milled powders are shown in Figure 1c. As presented in Figure 1c, only the characteristic diffraction peaks of W, Ni and Mn can be observed. After the mechanical milling, the phase compositions did not change, and the diffraction peaks of the composite powders became weaker and the peak width became larger. It should be noted that the mechanical milling process not only refines the powders but also causes significant lattice strain and, hence, increases the dislocations in the powder crystals [17]. The size distribution curves of the composite powders are illustrated in Figure 1d. Compared with the initial powders, the average particle size of the mechanical milling powders is much smaller, which is related to the mechanism of mechanical milling. Based on the above discussion, the powders were refined and the sintering activity of the initial powders were improved after mechanical milling, which is conducive to the preparation of fine-grained W-Ni-Mn alloys.

### 3.2. Densification Behavior of W-6Ni-4Mn Alloys

**Figure 2:**
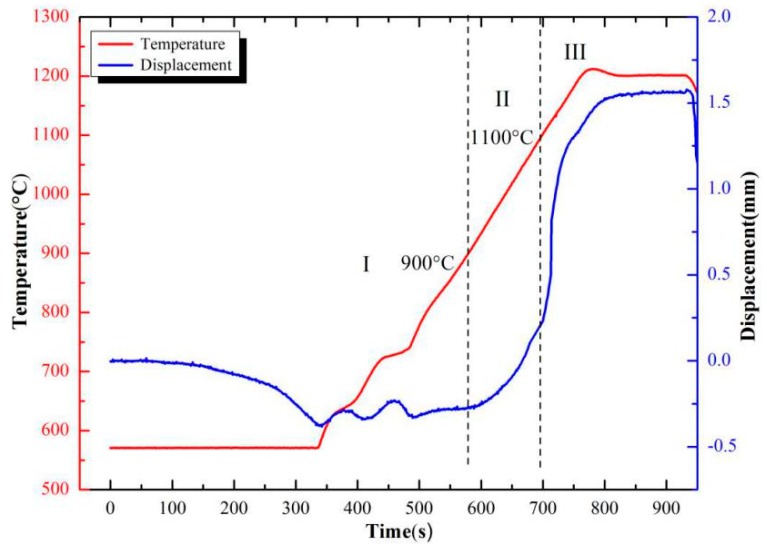
Curves of temperature and displacement vs. time in sintering process.

Figure 2 shows the sintered W-6Ni-4Mn alloy compacting displacement and temperature change with time. It is well known that liquid phase sintering can be divided into three stages: liquid phase generation and particle rearrangement stage, solution–reprecipitation stage, solid skeleton formation and grain growth stage [18]. Analogously, the sintering curve in Figure 2 could be divided into three stages, namely, the unshrinking stage, the solid state sintering stage and the instantaneous liquid phase sintering stage. In the unshrinking stage, sintering temperature ranges from room temperature to 900 °C, and the axial displacement of the powder compact slightly increases. In this stage, the degree of densification is small, mainly because of particle rearrangement being restricted at low temperature. As such, Ni and Mn cannot form the liquid phase. In the solid state sintering stage, temperature ranges from 900 to 1100 °C, and the axial displacement of the powder compact increased rapidly. In this stage, solid-state diffusion dominates the whole sintering process. As temperature rises, the increasing of the powder particles activation energy and inter-diffusion between Ni and Mn atoms result in the axial displacement fleetly increase. In the instantaneous liquid phase sintering stage, temperature ranges from 1100 to 1200 °C, the axial displacement increases exponentially in this stage, and the compact powders undergo rapid densification. Meanwhile, the γ-(Ni, Mn) binding phase generated and filled the pore among W powders by viscous flows, leading to rapid densification of the compact, so that a sharp rise in displacement. Finally, the sintering process enters the heat preservation stage. The displacement has almost no change observed from Figure 2, implying the nearly complete densification W-6Ni-4Mn alloy being obtained.

**Figure 3:**
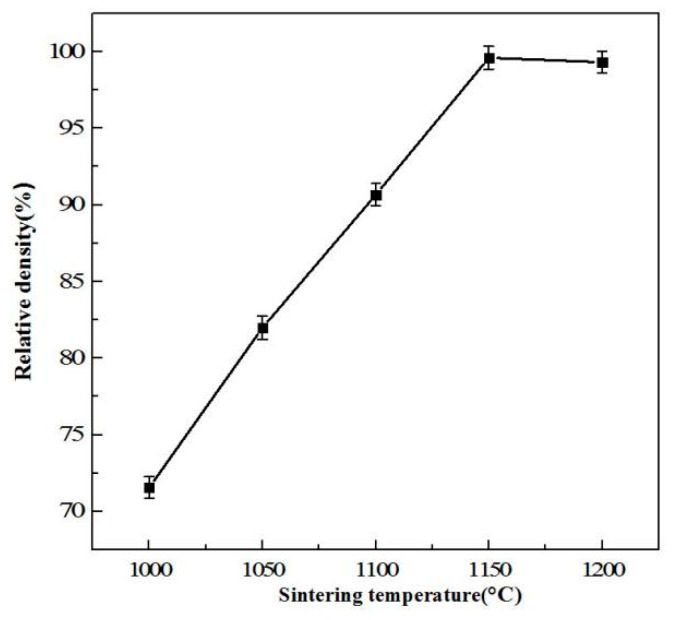
Relative density as a function of sintering temperature of W-6Ni-4Mn alloys.

**Figure 4:**
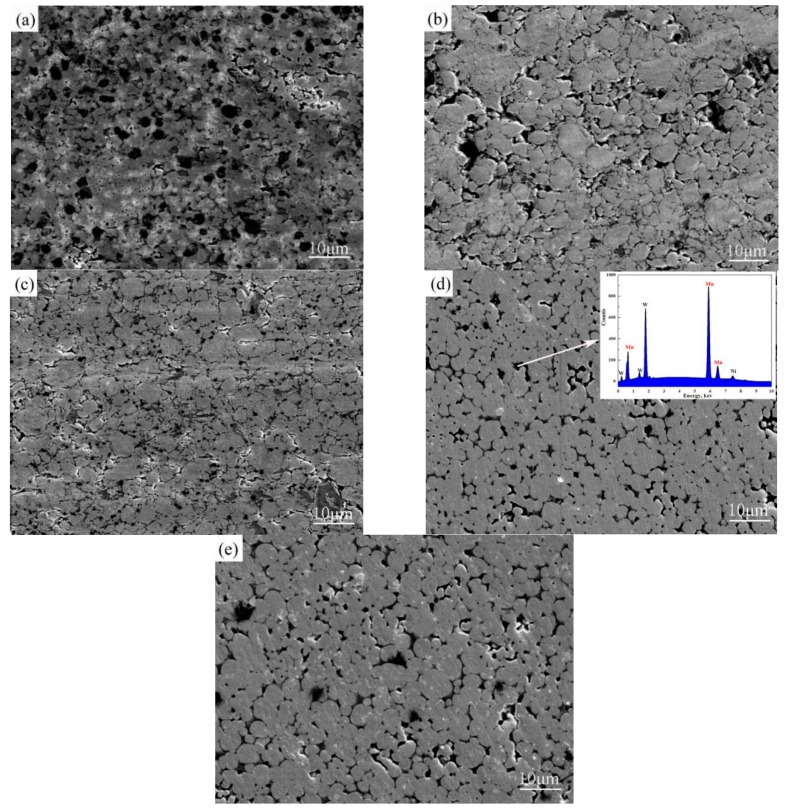
Cross-section morphology of W-6Ni-4Mn alloys at different sintering temperatures: (**a**) 1000 °C, (**b**) 1050 °C, (**c**) 1100 °C, (**d**) 1150 °C (inset EDS energy spectrum), and (**e**) 1200 °C.

Figure 3 illustrates the relative density as a function of sintering temperature of W-6Ni-4Mn alloy. When the temperature is below 1150 °C, the relative density increases rapidly as sintering temperature increases; the relative density then reaches the maximum density of 99.6% at 1150 °C, and the densification slightly decreases at 1200 °C. The binary phase diagram of Ni and Mn demonstrates that the temperature of the γ-(Ni, Mn) liquid phase formed by Ni and Mn is 1080 °C when the ratio of Ni to Mn is 6:4 [19]. When the sintering temperature is below 1050 °C, the liquid phase sintering did not begin, therefore, the alloy contains many micropores (Figure 4a,b). The relative densities at 1000 and 1050 °C can only reach 71.56% and 81.98%, respectively. When the sintering temperature increases to 1100 °C, the binding phase (Figure 4c) filled the micropores among W particles; therefore, the densification increases rapidly. However, 1100 °C is slightly higher than the liquid phase point of 1080 °C, but the sintered alloy has completed preliminarily densification. Thus, densification can reach to 90.66%. As sintering temperature further increases, the binding phase constantly filled the micropores by viscous flows and small W particles dissolved in the binding phase then precipitated on the surface of big W particles (Figure 4d,e). The densification reaches the maximum value of 99.6% at 1150 °C. When the temperature rises to 1200 °C, W grains grow in size, and the residual gas forms a closed internal porosity which hinders the densification increase. As a result, densification no longer increases or even slightly decreases.

### 3.3. Microstructure of W-6Ni-4Mn Alloys

Figure 4 shows the surface morphology of W-6Ni-4Mn alloys at different sintering temperatures. When the sintering temperature is below 1100 °C, due to agglomeration of starting powders, the W grain size increases slightly, the average grain size of W ranges from 3.1 to 4.5 µm, and the morphological characteristics of W particles did not appear as a typical indication of liquid phase sintering; most of them were combined together, leading to larger W grain size, and we found that the surface of the sintered alloy has a lot of micropores (Figure 4a,b). As sintering temperature increases, the liquid-phase sintering stage begins, the γ-(Ni, Mn) binding phase is formed, and small W particles are dissolved into the binding phase. When the dissolution enters a state of saturation, some small W particles precipitate on the large W particles from the binding phase, resulting in the increasing of W grain size, the shape of the W particles appears nearly spherical [18]. Furthermore, as the temperature increasing, the constant filling of the micropores by liquid phase leads to the W–W connectivity decreases, the grain size increases, and the sintered alloys are almost not porous. Some small black zones are also observed in Figure 4d–e. According to the EDS energy spectrum inserted in Figure 4d, the black zones are the Mn-rich phases rather than micropores. Figure 4 illustrates that the average grain size of the alloy increases from 3.1 (Figure 4a) to 6.5 µm (Figure 4e) as sintering temperature increases, which shows that the W grain size grows as sintering temperature increases. As shown in Figure 4, at a lower sintering temperature, the W grains formed a close link, leading to W–W connectivity being higher and the distribution of binding phase is scratch. As sintering temperature increases, the liquid phase filled the micropores among W grains constantly, resulting in W–W connectivity decrease and the binding phase well distribution. Above 1100 °C, the sintering temperature is closed to the theory formation temperature of the γ-(Ni, Mn) liquid phase, and liquid-phase sintering begins, so the W particles gradually become nearly spherical or spherical. When the sintering temperature is 1150 °C, the measurement value of W grain size approximately reaches 5.5 µm, the W–W connectivity is low, and the binding phase is uniformly distributed. Next, TEM was further employed to study the microstructure of the alloy sintered at 1150 °C.

**Figure 5:**
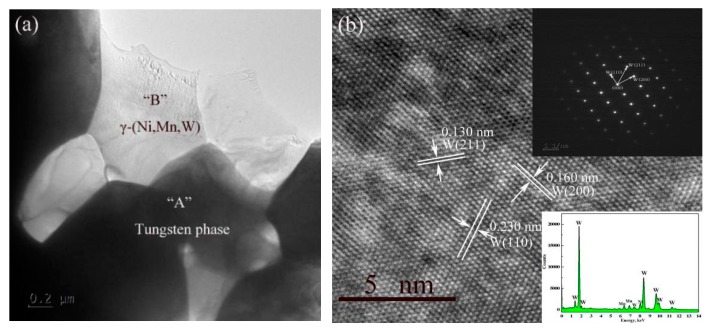

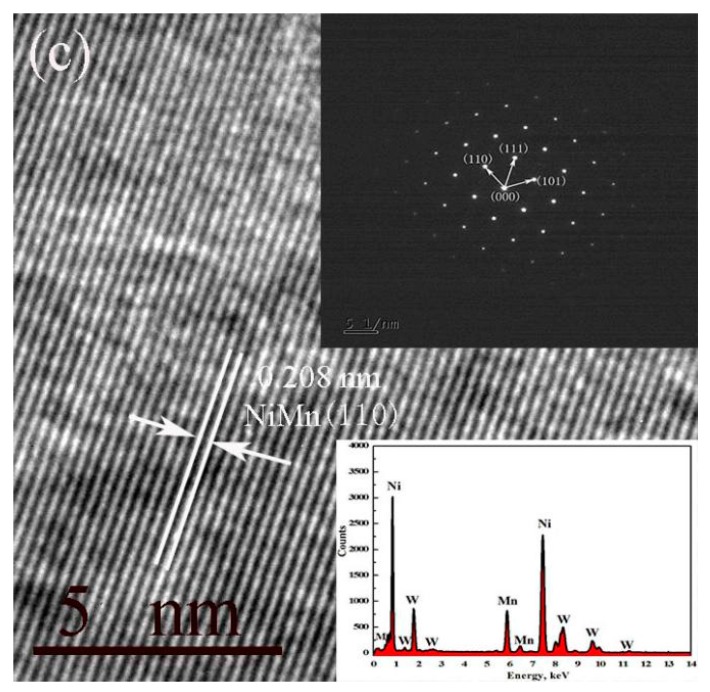
TEM investigation of the W-6Ni-4Mn alloy sintered at 1150 °C: (**a**) bright field image, (**b**) SEAD pattern and EDS energy spectrum of tungsten phase, and (**c**) SEAD pattern and EDS energy spectrum of γ-(Ni, Mn) phase.

Figure 5a shows the TEM bright field image of the W-6Ni-4Mn alloy. As illustrated in Figure 5a, point A was revealed as a dark area is the tungsten phase. The selected area diffraction (SEAD) pattern of the tungsten phase was displayed in Figure 5b. Based on the diffraction pattern, the tungsten phase has a BCC structure with the lattice parameters of 0.230 nm, 0.160 nm, 0.130 nm, which are consistent with the W (110), W (200) and W (211) crystal plane respectively. EDS analysis indicated that a few Ni and Mn atoms were dissolved in the tungsten phase during sintering. Figure 5a also shows the γ-(Ni, Mn) phase revealed as a bright area (see point B). According to the analysis of the SEAD pattern and EDS energy spectrum, as shown in Figure 5c, the spacing of the lattice fringe is 0.208 nm which is consistent with the Ni/Mn (110) crystal plane; meanwhile, some W atoms were also dissolved in the γ-(Ni, Mn) phase during sintering.

### 3.4. Mechanical Properties and Fracture Morphology of W-6Ni-4Mn Alloys

**Figure 6:**
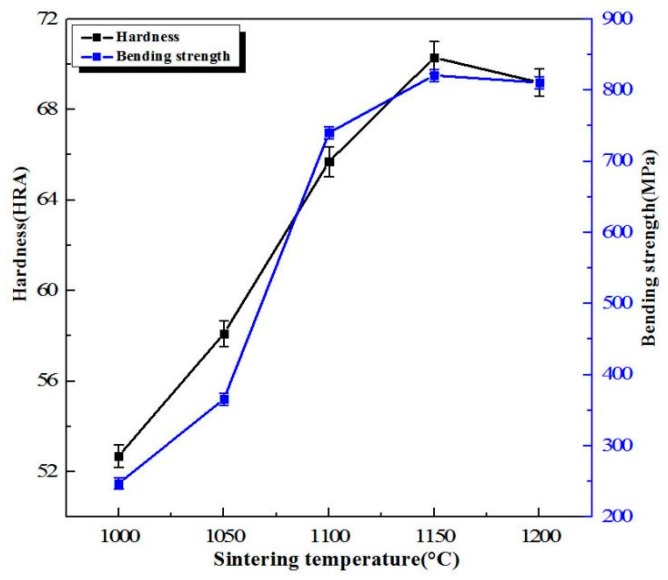
Hardness and bending strength of W-6Ni-4Mn alloys at different sintering temperatures.

Figure 6 shows the hardness and bending strength of W-6Ni-4Mn at different sintering temperatures. When the sintering temperature increases, hardness and bending strength initially increase and then decrease. Hardness is related to the characteristic of a specific material and its microstructure. For WHAs, the main factors influencing bending strength include W grain size and shape, W–W connectivity, the distribution of binding phase, the strength of W-binding phase and binding phase [20]. When temperature is below 1150 °C, with increasing the sintering temperature, the hardness and bending strength rapidly increase from 52.7 to 65.7 HRA and from 247.24 to 740.10 MPa, respectively. When the sintering temperature is 1150 °C, the hardness and bending strength reach the maximum values of 70.3 HRA and 820.90 MPa, respectively. A further increase in the sintering temperature causes a slight decrease in hardness and bending strength. The reason is that when the temperature is below 1150 °C, the densification of the sintered alloys is low and W–W connectivity is higher. This is attributed to the fact that binding phase is not formed or is formed but is unevenly distributed at a low sintering temperature. As the sintering temperature increases, the densification increases, W grains grow, the binding phase is uniformly distributed, and W–W connectivity decreases, resulting in the hardness and bending strength of WHAs increasing. When the sintering temperature continuously increases to 1200 °C, due to the higher temperature, the binding phase is easy to volatilize, thereby causing W–W connectivity increases, and W grain growth, leading to the decrease of bending strength. This result is also consistent with the densification curve of WHAs illustrated in Figure 3 and the microstructure shown in Figure 4. Mechanical milling starting powders can greatly reduce sintering temperature. These phenomena are possible because milling starting powders can improve the atomic activity and the dissolution of tungsten in the binding phase, and shorten the distance between diffused atoms during sintering. Thus, sintering temperature can be reduced.

**Figure 7:**
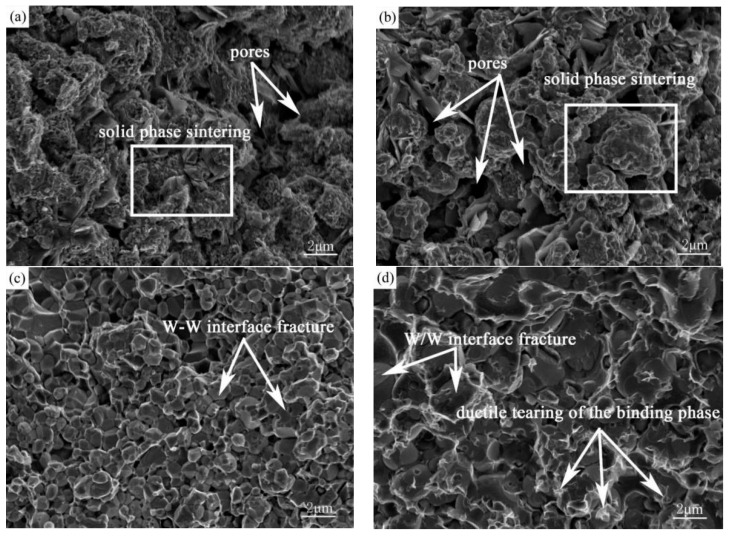

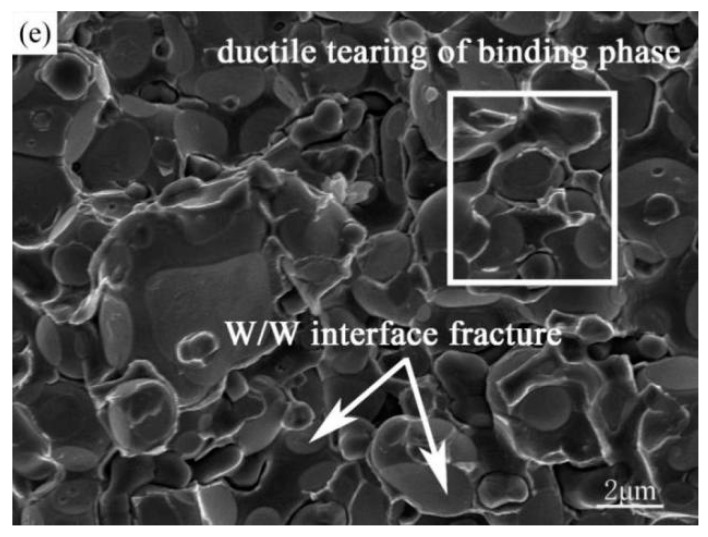
Fracture morphology of W-6Ni-4Mn alloys at different sintering temperatures. (**a**) 1000 °C, (**b**) 1050 °C, (**c**) 1100 °C, (**d**) 1150 °C, and (**e**) 1200 °C.

The fracture modes of WHAs are generally classified into four kinds: W/W interface fracture, W grain transgranular dissociation interface fracture, W–binding phase interface fracture, and ductile tearing of the binding phase [21]. The bending fracture morphological characteristics of W-6Ni-4Mn alloys sintered at different sintering temperatures are shown in Figure 7. When sintering temperature is below 1050 °C, the fracture is resulted from monolithic peeling-off along the section (Figure 7a,b), leading to a low bending strength. As temperature is further increased to 1100 °C (Figure 7c), the main fracture modes is W/W interface fracture. When the sintering temperatures are 1150 °C (Figure 7d) and 1200 °C (Figure 7e), the more binding phases are formed with the sintering temperature increasing, and thus, the ductile tearing of the binding phase is also observed in addition to intergranular fracture of the W grains, resulting in the improvement of the bending strength of the sintered alloys. The main fracture mode of W-6Ni-4Mn alloys is W/W interface fracture.

## 4. Conclusions

The W-6Ni-4Mn alloys with a high densification of 99.6% are fabricated by SPS at 1150 °C for 3 min. The sintered alloys are characterized by a small W grain less than 7 µm, and the binding phase distribution of the alloys is homogeneous. TEM analysis of the alloy shows that it is mainly composed of the tungsten phase and the γ-(Ni, Mn)-binding phase. As the sintering temperature increases, the hardness and bending strength of the alloys initially increase and then decrease. The best comprehensive mechanical properties of the alloy are attained at 1150 °C, and the hardness and bending strength are 70.3 HRA and 820.90 MPa, respectively. The fracture morphological characteristics of the alloy indicate that the main fracture mode is W/W interface fracture.
